# Neuroprotective effects of Bhilawanol and Anacardic acid during glutamate-induced neurotoxicity

**DOI:** 10.1016/j.jsps.2021.07.011

**Published:** 2021-07-15

**Authors:** Fadwa Al Mughairbi, Rukhsana Nawaz, Faisal Khan, Amina Hassan, Nailah Mahmood, Heba Tageldeen Ahmed, Alia Alshamali, Sagheer Ahmed, Asma Bashir

**Affiliations:** a Dep. of Clinical Psychology, College of Medicines and Health Sciences, UAE University, Al Ain, United Arab Emirates; b Panjwani Center for Molecular Medicine and Drug Research (PCMD), International Center for Chemical and Biological Sciences, University of Karachi, Karachi 75270, Pakistan; c Shifa College of Pharmaceutical Sciences, Shifa Tameer-e-Millat University, Islamabad, Pakistan; d Endodontic Department, Hamdan Bin Mohammed College of Dental Medicine, Mohammed Bin Rashid University of Medicine and Health Sciences, Dubai, United Arab Emirates

**Keywords:** Anacardic acid, Bhilawanol, Semecarpus anacardium, MTT assay, LDH assay, Caspase-3, Bcl-2

## Abstract

Bhilawanol (Bh) and anacardic acid (AA) are two lipid-soluble compounds mostly found in the nut of Semecarpus anacardium (SA). This herb has many medicinal properties including enhancing learning and memory, yet its active compounds have not been studied for neuroprotective effects. We investigated the neuroprotective effects of Bh and AA against glutamate induced cell death in the adrenal pheochromocytoma cell line of rats (PC12 cells). Cell viability, toxicity and calcium influx were determined by MTT assay, LDH release assay and Fluo-3 imaging while apoptosis was assayed by caspase-3 and Bcl-2 gene expression. Our results showed that Bh and AA treatments significantly increased cell viability, reduced cell toxicity and calcium influx in PC12 cells in addition to suppressing the reactive oxygen species. Furthermore, AA treatment decreased caspase-3 expression level whereas both Bh and AA enhanced the expression of anti-apoptotic gene Bcl-2 in PC12 cells. Both compounds potently inhibited acetylcholinesterase enzyme (AChE) in a dose and time dependent manner. These findings suggest that the traditional use of SA may be explained on the basis of both Bh and AA showing neuroprotective potential due to their effects on enhancing cell viability, reducing cell toxicity most probably by reducing excessive calcium influx and suppression of ROS as well as by decreasing the expression of proapoptotic caspase 3 gene and increasing the expression of antiapoptotic gene Bcl2. Traditional use in enhancing learning and memory was justified in part by inhibition of AChE.

## 1 Introduction

Herbs are highly valued because they contain active ingredients as sources of medicinal compounds and play important roles in maintaining and improving human health. Semecarpus anacardium L. (Anacardiaceae) (SA) commonly known as Bhallataka or marking nut is used in indigenous Indian, African, and Arabic systems of medicine for the treatment of various diseases (King, 1957). It is found in northern parts of India and grows naturally in tropical and dry climate (Vinutha et al., 2007). The nut has been found to be effective against diseases such as arthritis, and infections (Semalty et al., 2010). It is also used traditionally for dyeing hair, and for promoting hair growth (Vinutha et al., 2007). Furthermore, the nut extract has anti-tumor properties which is due to the suppression of hypoxic and angiogenic factors (Mathivadhani et al., 2007). There is an abundance of anecdotal evidence for the role of SA in enhancing learning and memory (King, 1957). Central nervous system benefits from the extract of either the whole plant or from the nut only, have been reported (King, 1957). However, the underlying mechanisms of these effects are not known. Early Arabs used SA nuts’ extract (known by them as Blather) to help them memorize Quran. It was believed that low doses of the extract improve memory, whereas high doses lead to psychotic symptoms. SA is also considered to have neuroprotective activity, and thus useful in neurodegenerative diseases like Alzheimer’s disease (AD). In addition, there has been a revival of interest in the medicinal plants recently for possible use in neuropharmacology which has prompted us to investigate the scientific rationale of the use of SA for enhancing learning and memory, and related neuroprotective effects.

Bhilawanol (Bh) and Anacardic Acid (AA) are two of the most important phytocompounds isolated from Semecarpus anacardium (SA). AA is a phenol lipid and bioactive phytochemical that is generally found in the cashew apple and nut in addition to SA. It is made up of 3-n-pentadecylcatechol and 3-n-pentadecenylcatechols (Kubo et al., 2006). Due to its cytotoxic activity against several tumour cell lines, as well as antibacterial and other inhibitory activities (Kubo et al., 2006), AA is being considered a potential target for treating serious diseases such as cancer, oxidative damage, obesity and inflammation (Hemshekhar et al., 2012). Bhilawanol (Bh) is one of the most significant and active ingredients of SA (Semalty et al., 2010). Bh, an allergenic oil, is considered highly effective against many diseases like arthritis, tumors and infections (Indap et al., 1983). Chromatography, nuclear magnetic resonance, and infra-red results has shown that Bh is a mixture of cis and trans isomers of ursuhenol and consists of 32% 1,2-dihydroxy-3-pentadecenylbenzene and 68–70% of the corresponding diene analogue (Rao et al., 1973, Gedam et al., 1974). There is a dearth of pharmacological information about this important phytocompound. Although some studies have addressed the chemical and physical properties of Bh and its poisoning, there are not many studies which have explored the pharmacological effects of Bh, especially in neurodegenerative diseases.

Both Bh and AA have ability to cross blood brain barrier but their beneficial effects of these compounds on neuron are not known yet. Therefore, this study was designed to investigate the protective effects of these two phytocompounds in excitotoxicity model induced by glutamate on PC-12 cells.

## 2 Methods and materials

### 2.1 Chemicals and other reagents

Rat adrenal pheochromocytoma (PC12) cells were used for this study. The cell line was obtained from ATCC. Bh and AA were purchased from Sigma (cat.# 122,228,198 and SH300341 respectively). RPMI, FBS and L-glutamic acid monosodium (*l*MSG) were purchased from Sigma-Aldrich Co. Lactate dehydrogenase (LDH) and 3-(4,5-dimethylthiazol-2-yl)-2,5-diphenyltetrazolium bromide (MTT) reagents were provided by Promega. Trypan Blue solution, L-glutamin, penicillin and streptomycin were obtained from HyClone U.S. MK801 blocker was purchased from Sigma-Aldrich, Germany. RNA Miniprep Kit was purchased from Promega, USA, and RT-PCR from Norgen Biotek, Canada. Acetylthiocholine iodide (ATCI), butyrylthiocholineiodide (BTCI) and all other reagents used for the current study were purchased from Sigma–Aldrich (USA).

### 2.2 Preparation of compound and reagents

100 ml of serum-free media was prepared by diluting 1 ml of antibiotic in 99 ml of RPMI. A stock solution of 100 mM was first prepared by dissolving 0.187 g of glutamate in 10 ml serum-free media. From this stock, a concentration of 20 mM was chosen to be used to cause cell damage before treating with the Bh and AA. 1 µM stock solution of Bh and AA Acid were prepared by diluting 1 mM Bh and AA in serum-free media respectively. The different concentrations of Bh and AA (25 nM, 50 nM, 100 nM and 200 nM) were prepared using this stock.

### 2.3 Cultivation of PC12 cell line and treatment

The required amount of glutamate was first dissolved in fresh medium and then pH adjusted to 7.0 and filtered through a 0.2 μm filter. The fresh filtrate was used for further analyses. Bh and AA were dissolved in DMSO and stored at –20 °C. The stock solution was further diluted with the fresh medium to obtain different concentrations of test solutions. PC12 rat adrenal pheochromocytoma cells were obtained from ATCC and cultured in complete media (RPMI + 10% FBS + 1% penicillin/streptomycin), replaced with new media every 2–3 days, and incubated at 37 °C under 5% CO_2_ atmosphere. Cells were seeded into 96-well plates with a density of 15,000cells/well. Three different plates were prepared for each of the treatments to treat cells for 24 and 48. Cells were incubated at 37 °C under 5% CO_2_ atmosphere to adhere for approximately 24 h before treatment. The medium was changed with one that contained 20 mM of glutamate. Concentration of 0 was considered as control. To see the protective effect of compounds, we added different concentrations (25 nM, 50 nM, 100 nM and 200 nM) of both Bh and AA in each well plate and each concentration was used in triplicate.

### 2.4 MTT assay

MTT proliferation assay kit was used to see the effect of compounds on cell viability as previously described (15 and 16). PC12 cells were grown in 96 well tissue culture plates and treated with both compounds. After incubating for a specific time period, culture media was removed and 10μlof MTT solution (5 mg/ml) was added to each well and incubated for 4 hr. Then 100 μL of DMSO was added. Absorbance was recorded at a wavelength of 570 nm using ELISA reader. The cell viability (%) was calculated as follows:

O.D of treated wells − O.D of blank wells

-------------------------------------------------X 100.

O.D of control wells − O.D of blank wells

### 2.5 LDH assay

The toxic effect of glutamate on PC12 cells was also determined by lactate dehydrogenase (LDH) release assay (Thermo Scientific) as per the manufacturer’s instructions. PC12 cells were treated with L-glutamate and Bh and AA at different concentrations for 24 and 48 hrs. After treatment, cells were spun down by centrifugation at 2500 g for 5 min at 4 °C. Supernatant of cells (50μl) was transferred to a new plate and 50ul of reaction mixture was added and incubated for 30 min at room temperature. 50ul of stop solution was then added and absorbance was measured at 490 nm and 492 nm wavelengths. Toxicity was calculated by the following formula:

Experimental LDH Release (OD490)

Percent cytotoxicity = ---------------------------------------------- X 100

Maximum LDH Release (OD490)

### 2.6 Determination of Intra-cellular reactive oxygen species

The method for the determination of total intracellular reactive oxygen species (ROS) was carried out using kit. The untreated sample was taken as the control. The determination was repeated with triplicate samples and the total ROS were calculated.

### 2.7 Intracellular calcium detection

The concentration of intracellular Ca^+2^ was measured with Fluo-3 AM by the method followed by Aoshima et al. (Aoshima et al., 1997), with some modifications. Briefly after the treatment, cells were washed and fresh buffer solution was added (containing 140 mM NaCl, 5 mMKCl, 1 mM MgCl_2_,5.6 mM glucose, 1.5 mM CaCl_2_, 20 mM HEPES (pH 7.4), Fluo-3 AM (5 µM)), and subsequently incubated for 30 min at 37 °C. After washing three times, fluorescence intensity of Fluo-3 was quantified by a fluorescence spectrophotometer – (Synergy HTX) at an excitation wavelength of 480 nm and an emission wavelength of 526 nm. [Ca^+2^]i was calculated from the Fluo-3 fluorescence intensity using the equation:

[Ca^+2^]i = Kd (F0-Fmin)/(Fmax-F0)(nmol/l).

For the purpose of calculation of [Ca^+2^]i, the Kd was assumed to remain constant between 10 and 25 °C, and increase linearly up to 42 °C and value is 400 nmol/l at 25 °C. The maximal Fluo-3 fluorescence intensity (Fmax) was determined by adding 0.1% Triton X-100 and the minimal fluorescence (Fmin) was determined by quenching Fluo-3 fluorescence with 5 mM EGTA. F0 is the fluorescence measured without Triton X-100 or EGTA.

### 2.8 Acetylcholinesterase assay

The inhibitory activities of the extract of SA on AChE enzyme were measured by Ellman’s method and as described previously (Adhami et al., 2011). To preparation rat erythrocytes membrane, the blood (2.5–3 ml) was drawn from the rat and transferred to 50 ml de-ionized chilled water. After two hours, the blood was centrifuged at 1500 g for 10 min. The supernatant was discarded and pellet was passed through 27 gauge syringe needle, followed by addition of lysis buffer. After 15 min, centrifugation was conducted (250 g) and pellet was washed with de-ionized water. The alternating process of adding lysis buffer and de-ionized water to the pellet was continued until a clear pellet was achieved, which was re-suspended in 1 ml of buffer containing Triton X (0.1%).

In a 96-well plate, 25 µL of 15 mM ACTI, 125 µL of 3 mM DTNB, 50 µL of buffer and 25 µL of extract solution were mixed. After 5 min, absorbance was measured at 405 nm. Then 25 µL of AChE (from membrane preparation) was added and the plate was incubated at 25 ◦C for 10 min. Then the absorbance was measured again every 5 min. A solution of 10% DMSO was used as negative control. The absorbance before addition of the enzyme was subtracted from the absorbance after adding the enzyme. The assay was performed with three repetitions for every concentration. Each experiment was carried out in triplicate.

### 2.9 Quantitative RT-PCR

PC12 cells (1X 10^8^) were cultured in T-75 flasks and treated with glutamate, glutamate in combination with MK801 blocker, glutamate with Bh and AA. After 24 h treatment total cellular RNA was extract by RNA Miniprep Kit method. The quality of extracted RNA was determined via spectrophotometer (260/280 nm). Equal amount of total RNA (1 µg) for control and all treated groups were transcribed to cDNA using SuperScript First Strand Synthesis System for RT-PCR. Real time quantitative RT-PCR (Q-RT-PCR) was performed on cDNA in the presence of a 1 × Master mix containing pre-set concentrations of dNTPs, MgCl_2_, Taq DNA polymerase and buffers. Target Genes primers were used for comparison and analysis; Caspase-3 gene primer set: (Forward primer;5′ACGGGACTTGGAAAGCATC3′; reverse 5′TAAGGAAGCCTGGAGCACAG3′); BCl2 gene primer set; (Forward primer; 5′GGTGGACAACATCGCTCTG3′;reverseprimer 5′CAGCCAGGAGAAATCAAACA3′); Beta actin Gene primer set; (Forward Primer5′ACGCATCCACCAAGAAGC3′; Reverse primer 5′ GCCACACGGAAGAAGACCT3′. After an initial denaturation at 94 °C for 4 min, 40 cycles were performed using the PCR condition: 94 °C, 30 s; 55 °C, 30 s; and 60 °C, 4 min and hold at 4 °C. Each sample of the targeted genes (Caspase-3 and Bcl2) expression was assayed in the duplicates and the samples were electrophoresed at 90 V. Gene expression was quantified and visualized by gel documentation system and images were documented.

### 2.10 Statistical analysis

ANOVA was done using statistical analysis system SPSS v. 20.0 (SPSS Inc., Chicago, IL, USA) software to analyze the variances. Duncan’s multiple range tests were used to test the significance of differences between paired means. Data were presented as mean ± standard error. The significance of the difference was determined by a confidence level of p < 0.05.

## 3 Results

### 3.1 Determination of compounds of SA

A search for the active compounds of SA through the Natural Product Dictionary resulted in finding 14 active compounds (supplementary, Table 1). ACD/I-Lab prediction algorithm suggested that only two of them are active in the brain. Therefore, we selected only Bh and AA for further investigation. To conform weather these compounds are present in the nut, we ran HPLC for the extract and compounds (supplementary Fig. 1).

### 3.2 Bh and AA improved glutamate compromised cell viability

**Fig. 1:**
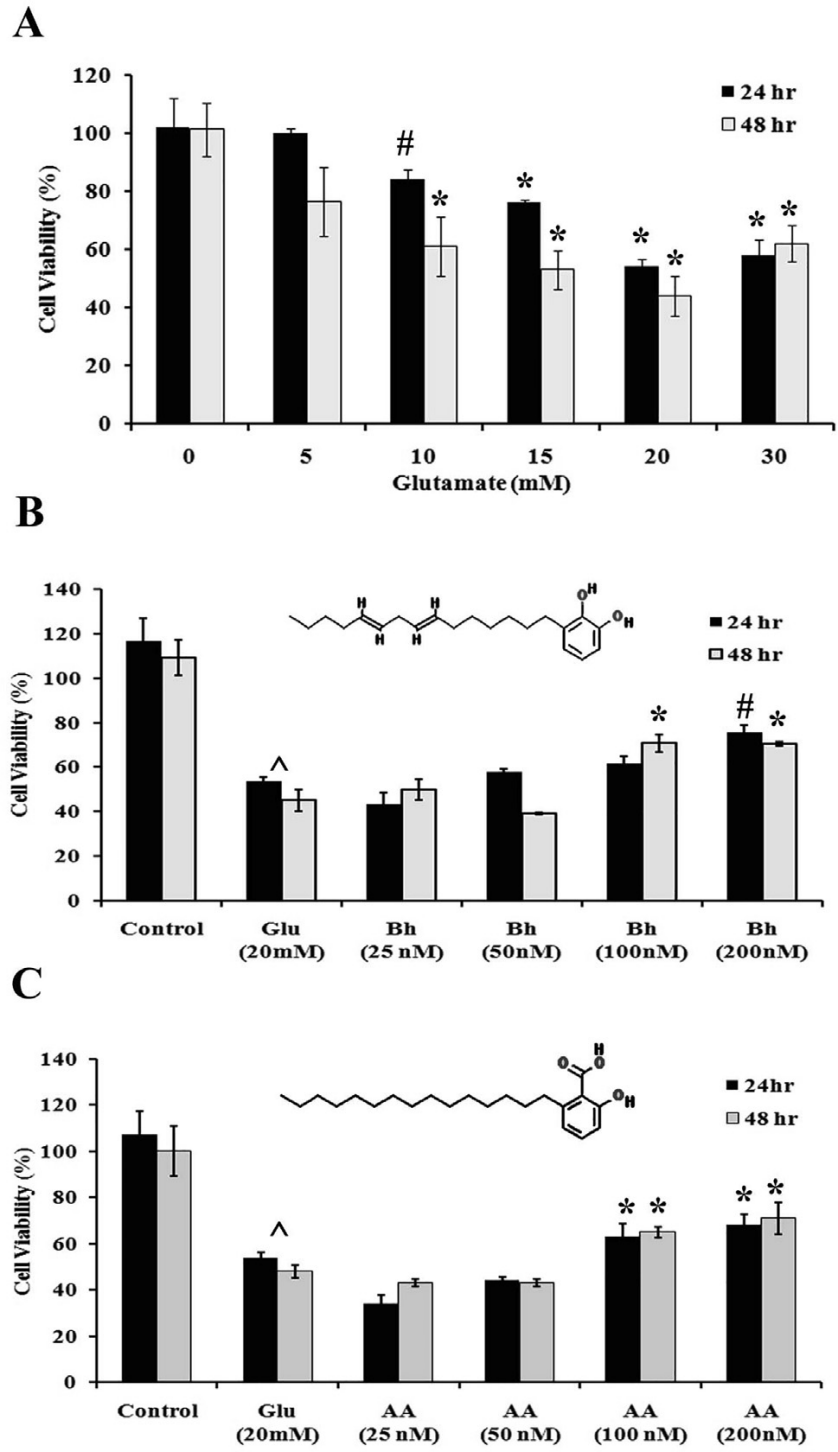
Effects of Bhilawanol and anacardic acid on PC12 cells viability against glutamate toxicity using MTT assay. A) Glutamate toxicity on cell viability. B) Protective effect of Bhilawanol and C) Anacardic acid. Inset showing chemical structure of compound. # p < 0.05 and * p < 0.01 when compared with Glu treated cells and **^** showed significance p < 0.001 between control and glutamate group.

PC12 cells were treated with glutamate (5, 10, 15, 20 and 30 mM concentration) for 24 and 48 h. Our results showed that the cell viability was decreased by glutamate in a concentration dependent manner (Fig. 1A). Glutamate at a concentration 20 mM showed about 50% inhibition of cell viability, therefore this concentration was chosen for the subsequent experiments. The viability of cells treated with 20 mM of glutamate was calculated at 53% and 57% in comparison to the control after 24 hr and 48 hr of treatment, respectively. Cell viability was significantly increased after incubation of glutamate treated cells with various concentrations of Bh and AA. Bh, at a concentration of 100 nM increased cell viability non-significantly from 53% to 55.3 after 24 hr but at 48 h’ mark, cell viability was increased significantly to 67.7 % (Fig. 1B). Bh caused a significant increase in the cell viability at 200 nM, from 57% to 70.6 after 24 h and to 72.88% after 48 h (Fig. 1B). There was a dose and time-dependent increase in the cell viability after pretreatment with various doses of AA (Fig. 1C). Although no significant increase in the cell viability was observed at 25 and 50 nM concentrations, cell viability increased significantly at 100 and 200 nM.

### 3.3 Bh and AA reduce cell toxicity

**Fig. 2:**
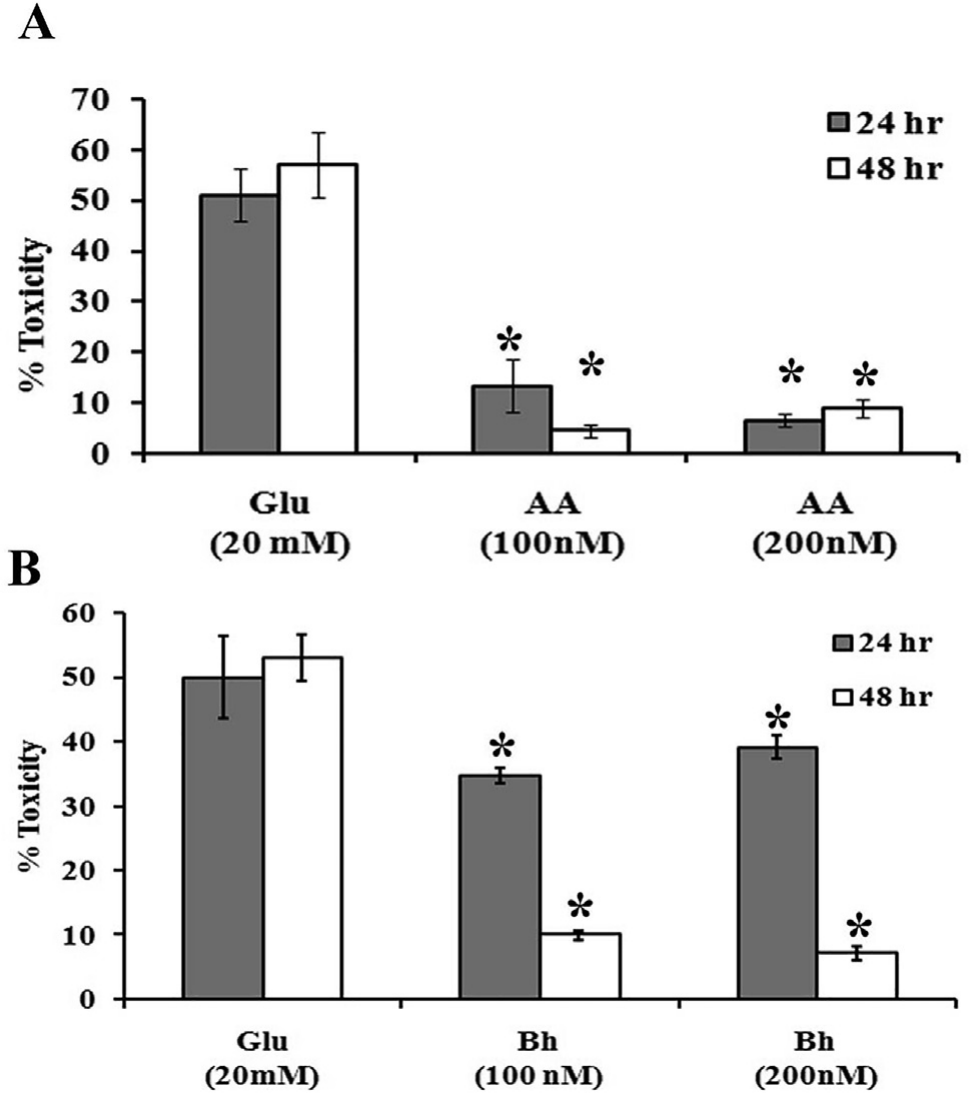
Effects of Bhilawanol (Bh) and anacardic acid (AA) on PC12 cell toxicity assessed by LDH assay.*p < 0.01 when compared with Glu treated cells.

To further investigate the protective effect of Bh and AA against glutamate-induced toxicity in PC12 cells, LDH assay was used. Treatment with glutamate (20 mM) resulted in more than 50% increase in cell toxicity compared with control. AA was more potent in reducing cell toxicity at 24 and 48 h after the dose of 100 and 200 nM (Fig. 2A). Incubation with Bh at 100 and 200 nM doses significantly decreased cell toxicity in the PC12 cell system to 34% and 39% respectively after 24 hr. After 48 h’ cell toxicity was even further decrease to 10% and 7.22% for 100 and 200 nM doses, respectively (Fig. 2B). However, at 24 h after the dose, the effect of Bh was less pronounced than that of AA, although still significant compared with the control (Fig. 2B). After 48 h, the effects of both compounds were comparable.

### 3.4 Glutamate induced production of ROS and calcium influx

**Fig. 3:**
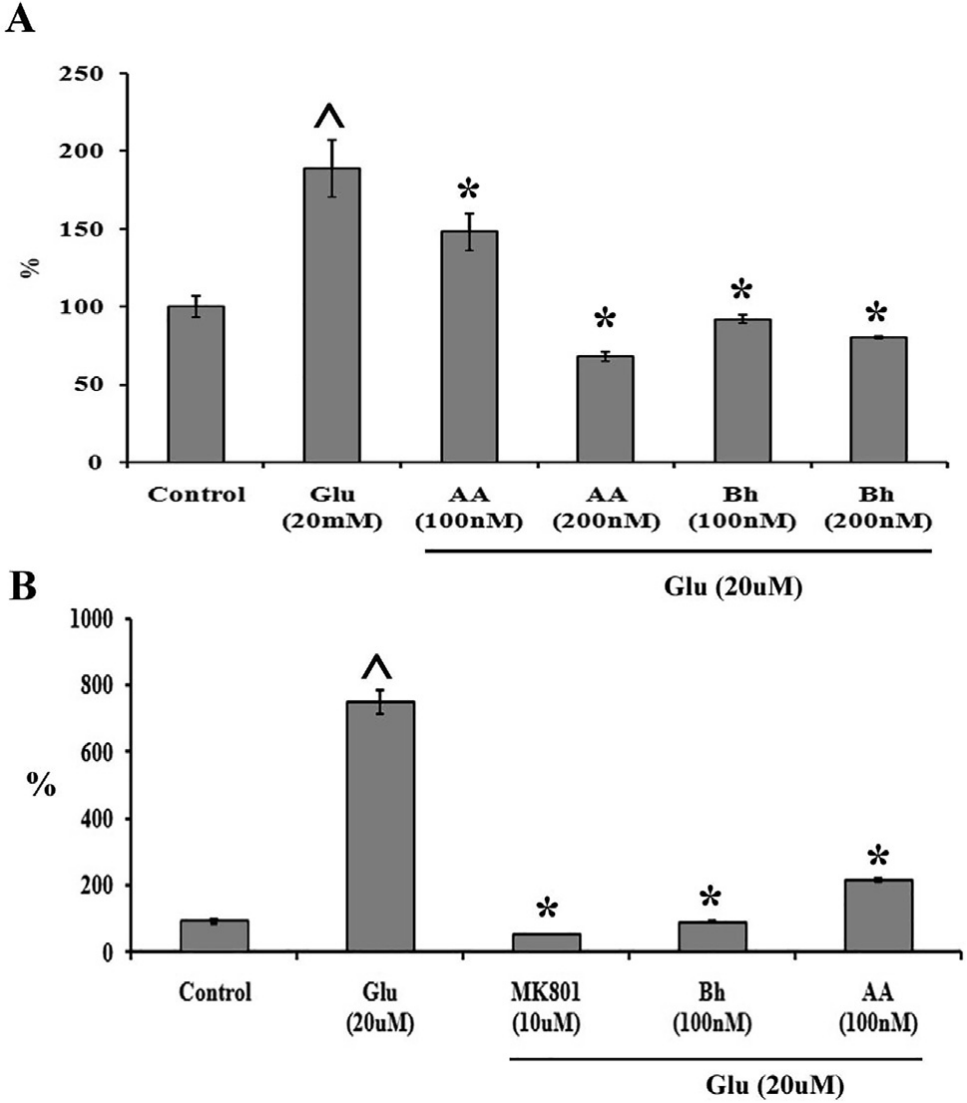
Effect of AA and Bh on ROS and Calcium influx**.** A) ROS level in PC12 cells were detected by DCF dye. B) Intracellular level of calcium in PC12 cells was detected by measuring fluorescence of Fluo-3 dye. **^** indicates the p < 0.001 compared with control and * p < 0.001 when compared with Glu treated cells.

The ROS production after glutamate exposure was increased (Fig. 3A). A significant increase in the dichlorofluorescein (DCF) dye was observed in the PC12 cells, 24 h after treatment with 20 mM glutamate. There was a 2-fold increase compared with control. However, pre-treatment with Bh and AA effectively reduced ROS generation. The ROS quenching effect demonstrated dose-dependency with the effect increasing with increasing concentrations of compounds (Fig. 3A).

Fluo-3AM fluorescent dye was used to measure calcium entry into PC12 cells which, if in large amounts, could be fatal for the cell. We investigated the effects of AA and Bh on whether they are able to prevent or reduce excessive calcium entry in the cell. Our results confirmed the protective effect of Bh and AA, showing a reduction in the calcium entry in the PC12 cells. The fluorescence intensity of Fluo-3AM which was increased several folds after glutamate treatment compared with control group, was brought down significantly by AA and Bh. AA, at 100 nM concentration was slightly more potent than Bh at the same dose. However, MK801, a competitive inhibitor of glutamate channels was the most effective compound in reducing calium entry in PC12 cells (Fig. 3B).

### 3.5 Acetylcholinesterase (AChE) assay

**Fig. 4:**
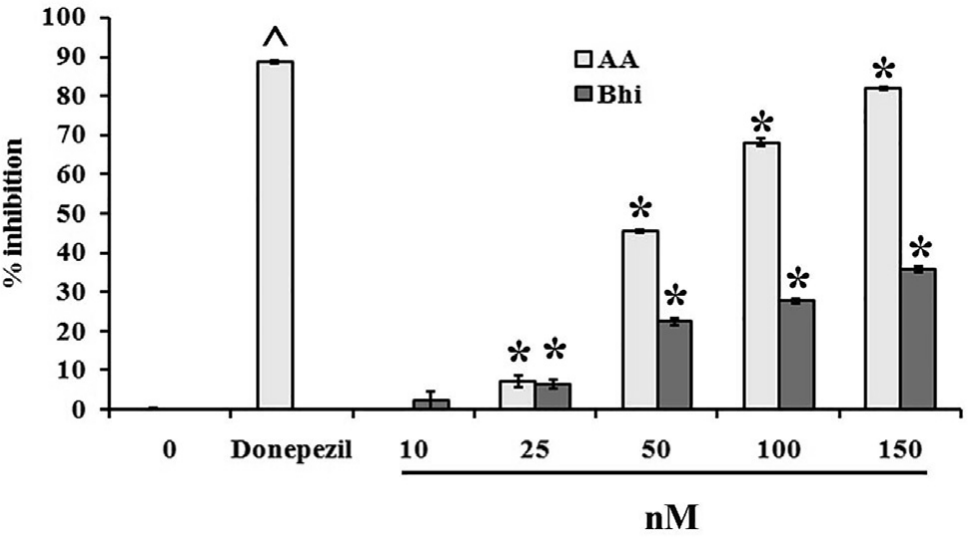
Bar graph represents % inhibition of compounds on AChE using Ellman’s method. **^** showed significance p < 0.001 compared to control and * indicated the p value < 0.001 as compared to the donepezil group.

The AChE inhibitory effects of Bh and AA were investigated in a microplate assay. Both compounds showed a dose-dependent inhibition of the enzyme with significant effects observed at 25, 50, 100, and 150 nM doses. AA was more potent than Bh. The IC50 values were 150 nM for Bh and 50 nM for AA. Donepizel, used as a standard for AChE inhibition, was found to be the most potent inhibitor in our assay (Fig. 4).

### 3.6 Apoptotic genes expression

**Fig. 5:**
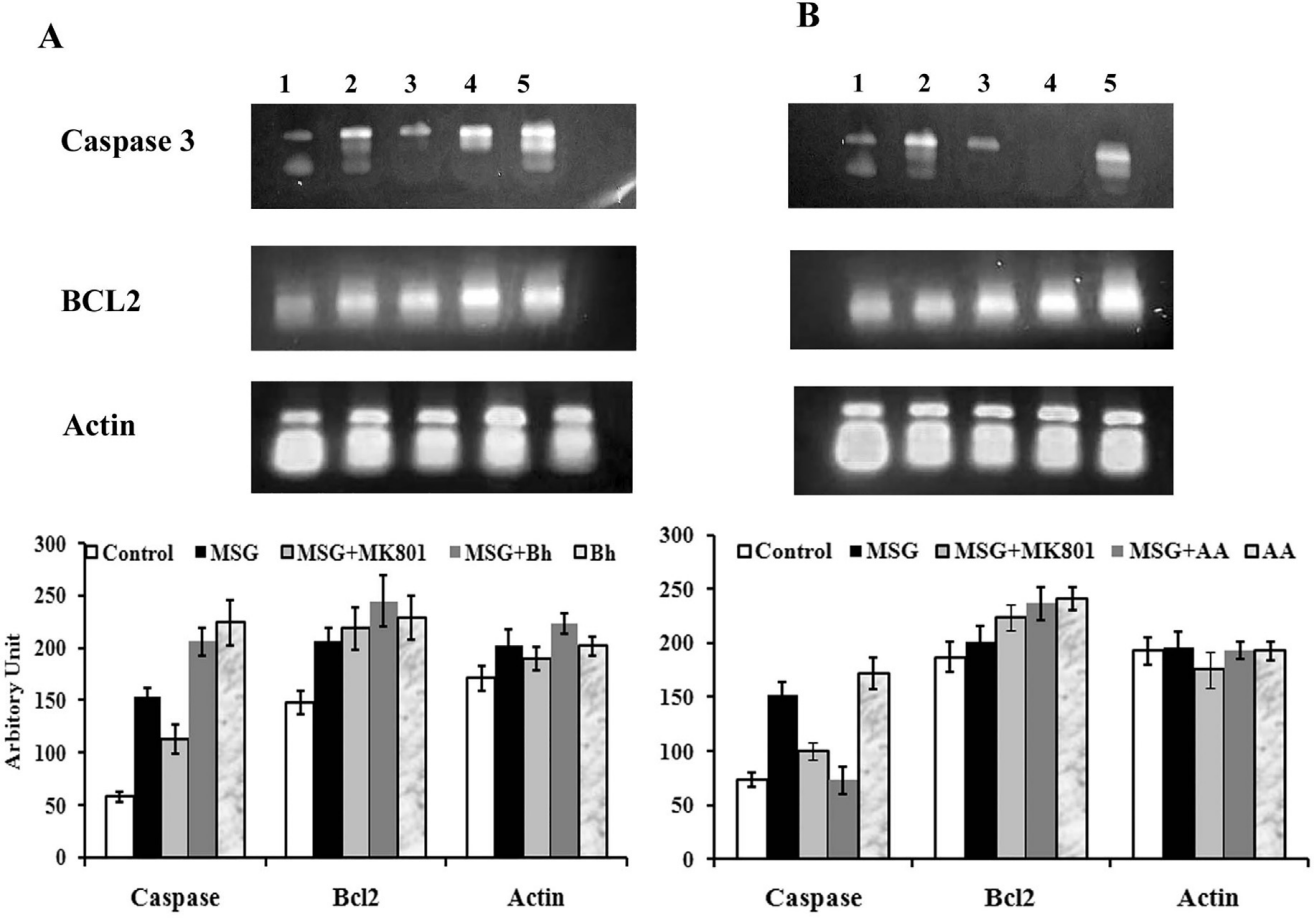
Expression of apoptotic genes in PC12 cells after treatment. A) Effect of Bh on gene expression. B) Effect of AA on Gene Expression. 1-Control, 2- Glutamate, 3-MK801, 4- Glutamate + Compound, 5- Compound alone.

Caspase-3 and bcl-2 genes expression was measured to find out whether AA and Bh protect PC12 cells from apoptosis. As shown in Fig. 5A, capase-3 expression significantly increased in glutamate treated group when compared with control group although expression reduced in MK801(NMDA blocker) treated group. Bh did not reduce caspase-3 expression but enhanced the Bcl2 expression at 100 nM (Fig. 5A). Moreover, the expression of caspase-3 was drastically reduced in PC12 cells treated with 100 nM AA. Bcl2 expression was increased by a 100 nM dose of AA (Fig. 5B).

## 4 Discussion

In the present study, we used a glutamate-induced toxicity model in PC12 cells to demonstrate neuroprotective effects of two of the most prominent but less studied phytocompounds from SA- AA and Bh. Glutamate-induced toxicity model is frequently used to explore neuroprotective effects of endogenous and exogenous compounds (Nampoothiri et al., 2014). L-glutamate, a principal excitatory neurotransmitter in the mammalian central nervous system (CNS), is associated with cognition, memory, and movement (Steckler et al., 1998). However, excess extracellular glutamate level could induce brain damage and over-activation of ionotropic glutamate receptor which induces neuronal death, through a pathway called excitotoxicity (Chen et al., 2002). High levels of extracellular glutamate could induce oxidative stress, contributing to neurodegenerative diseases by stimulating the generation of reactive oxygen species (ROS), mitochondrial hyperpolarization and lipid peroxidation in neuronal cells (Sanabria et al., 2002). Our results reveal that Bh, and more potently AA, offer neuroprotection through several different mechanisms.

Our results showed that the cell viability was drastically reduced by glutamate application in a concentration dependent manner which, however, was recovered by pretreatment with AA and Bh. This enhanced cell viability indicates neuroprotective potential of these phytocompounds. Cell toxicity as measured by the LDH assay also exhibited a significant effect of AA and Bh by reducing cell toxicity in glutamate treated PC12 cells. AA was more potent than Bh in protecting against cell toxicity. These two pharmacological effects seem to be the underlying mechanisms responsible for the observed neuroprotective effects of SA in traditional medicine, especially when many of the phytochemicals are known to enhance cell toxicity and reduce cell viability(Abdullah et al., 2014, BACANLI et al., 2017).

Since glutamate induced cell toxicity is mediated by excessive calcium influx and generation of ROS, we investigated the effects of AA and Bh on calcium entry in the cell and ROS quenching in order to find out whether increase in cell viability and reduction in the cell toxicity by these two compounds involve any effects on these cellular insults. Excess accumulation of ROS leads to cellular damage and inflammation of the tissues (Murphy et al., 2011). ROS plays a major role in cellular senescence paving way to neural cell death. Both the compounds demonstrated ROS quenching effect in a dose-dependent manner with the effect increasing with increasing concentrations of compounds. Similarly, glutamate induced calcium entry in the cell was significantly reduced by both the compounds, Bh being slightly less potent. These results suggest that neuroprotective effect by AA and Bh as observed by their effects on cell viability and toxicity may be in part due to their effect in reducing calcium entry in the cell and suppression of ROS generation. Several plants and compounds isolated from them have previously demonstrated similar properties, many of them possessing strong free radical scavenging activity, and preventing oxidant–induced cellular damage (G.M.Husain et al., 2007),

Cell viability at early stages of apoptotic cascade also depends on the balance between pro and anti-apoptotic factors (Cory and Adams, 2002). Caspase-3 which plays a major contribution in the execution of neuronal apoptosis, is markedly decreased during the process of brain maturation (Yakovlev et al., 2001)*.* Previous studies strongly indicate that suppression of apoptotic capability in the mammalian brain during the postnatal period coincides with marked down-regulation of caspase-3 expression. Our results indicate that pretreatment with AA significantly reduces the elevated caspase-3 gene expression in PC12 cells. Bh did not show any noticeable effect. On the other hand, both compounds increased Bcl2 gene expression suggesting events of cellular protection in glutamate induced excitotoxic cells initiated by these compounds. These results are in agreement with previous studies where pretreatment of neuronal cells with a phytocompound caffeoylquinic impaired apoptosis by inhibiting the activation of caspase-3 and enhancing Bcl-2 expression(Tian et al., 2016).

Inhibitors of AChE enzyme are clinically used in Alzheimer’s disease to improve memory and cognition and are the only approved agents by the FDA for the disease in addition to mementine-a glutmate receptor antagonist. Their efficacy is due to the fact that AChE inhibitors improve the concentration of acetylcholine at synpases in the areas of the brain responsible for learning and storing memories as well as their retrieval, especially in hippocampus. Both AA and Bh exhibited a dose-dependent inhibition of the enzyme with significant effects starting at 25 nM dose with maximum effects at 150 nM dose. AA was more potent than Bh as indicated by the IC50 value of 50 nM for AA and 150 nM for Bh. This effect of the phytocompounds was similar to clinically available AChE inhibitors donepizel, rivastigmine, galantamine and tacrine. Several phytocompounds have previously reported similar effect (Mathew et al., 2014, Vladimir-Knežević et al., 2014, Osama et al., 2017).

We conclude that the traditional use of SA for neuroprotection and enhancement of learning and memory may be rationalized by the finding of our study. The two phytocompounds from SA-AA and Bh improved cell viability and reduced cell toxicity demonstrating scientific rational behind their neuroprotective role. The mechanism of action of these two phytocompounds seem to be inhibition of ROS and reduction in the excessive calcium influx in the cells, both of which may lead to apoptotic death of the neuronal cells. These compounds not only inhibit ROS generation and calcium entry, they also significantly reduce the expression of caspase 3 gene which is an important executive mechanism of apoptosis. Furthermore, enhancing the expression of anti-apoptotic gene Bcl2 may also play a role in the neuroprotection observed with AA and Bh. Both the compounds potently inhibited AChE which would explain its traditional use in enhancing learning and memory. Both phyto-compounds from SA, especially, AA which is more potent of the two, seems to have dual inhibitory mechanism-inhibiting AChE as well as glutamate. No drug is currently available which could inhibit both AChE and glutamate-the only two approaches targeted by the drugs currently approved for AD by FDA. Therefore, in addition to justifying the traditional use of SA for neuroprotection and enhancing learning and memory, these phytocompounds could be starting material for developing dual inhibitors of AChE and glutamate receptors.

## Declaration of Competing Interest

The authors declare that they have no known competing financial interests or personal relationships that could have appeared to influence the work reported in this paper.
