# Supplementary material for: Neuroprotective effects of Bhilawanol and Anacardic acid during glutamate-induced neurotoxicity
Source: Saudi Pharm J. 2021 Jul 15;29(9):1043–9. doi: 10.1016/j.jsps.2021.07.011 (PMC8463467; doi:10.1016/j.jsps.2021.07.011)
Supplement: Supplementary data 1 [file mmc1.docx]

**Supplementary Data:**

|  | **Chemical Name** | **Synonym** | **Formula** | **Solubility**  **(mg/ml)** | **BBB**  **(logBB)** | **Brain activity** |
| --- | --- | --- | --- | --- | --- | --- |
| 1 | 8,3'''-Bi[4',7-dihydroxyflavanone] | 8-[5-(3,4-Dihydro-7-hydroxy-4-oxo-2H | C30H22O8 | 3.2 | -0.74 | Inactive |
| 2 | Galluflavanone |  | C30H22O11 | 18.0 | -1.38 | Inactive |
| 3 | 3',4',4''',5,5'',7''-Hexahydroxy-8,3'''-biflavanone; (2*S*,2''*S*)-form |  | C30H22O10 | 3.7 | -0.73 | Inactive |
| 4 | 3',4',4''',5,5'',7''-Hexahydroxy-8,3'''-biflavanone; (2*S*,2''*S*)-form, 4',4''',7''-Tri-Me ether |  | C33H28O10 | 0.23ug/ml | -0.6 | Inactive |
| 5 | 3',4',4''',5,5'',7''-Hexahydroxy-8,3'''-biflavanone; (2*S*,2''*S*)-form, 3',4',4''',7''-Tetra-Me ether |  | C34H30O10 |  |  |  |
| 6 | 3',4',4''',5,5'',7''-Hexahydroxy-8,3'''-biflavanone; (2*S*,2''*S*)-form,  5''',7-Dimethoxy, 4''',7''-di-Me ether, 3',4'-methylene ether | Anacarduflavanone | C35H30O12 | 1.2ug/ml | -0.87 | Inactive |
| 7 | Jeediflavanone |  | C30H22O11 | 4.9 | -1.2 | Inactive |
| 8 | 5-Methyl-1,3-benzenediol; *O*-[β-D-Glucopyranosyl-(1→6)-β-D-glucopyranoside] | Anacardoside | C19H28O12 | 167 | -2 | Inactive |
| 9 | 3-Pentadecyl-1,2-benzenediol; 8',9'-Didehydro(*Z*-) | Bhilawanol A.  (15:1)-Urushiol. | C21H34O2 | 1.2 | 1 | Active |
| 10 | 3-Pentadecyl-1,2-benzenediol; 7',8',10',11'-Tetradehydro | Bhilawanol B | C21H32O2 | 1.9 | 1.1 | Active |
| 11 | Robustaflavone; 2*S*,2''*S*,3,3''-Tetrahydro |  | C30H22O10 | 3.3 | -1 |  |
| 12 | Semecarpetin |  | C34H30O9 | 0.28ug/ml | -0.54 | Inactive |
| 13 | Semecarpuflavanone |  | C30H22O10 | 9.9ug/ml | -1.24 | Inactive |
| 14 | 4',5,5''-Trihydroxy-3',4''',5',5''',7,7''-hexamethoxy-8,3'''-biflavanone | Nallaflavanone | C36H34O13 | 0.1ug/ml | -0.48 | Inactive |

**Table 1**: List of active compounds of SA with their solubility and brain activity


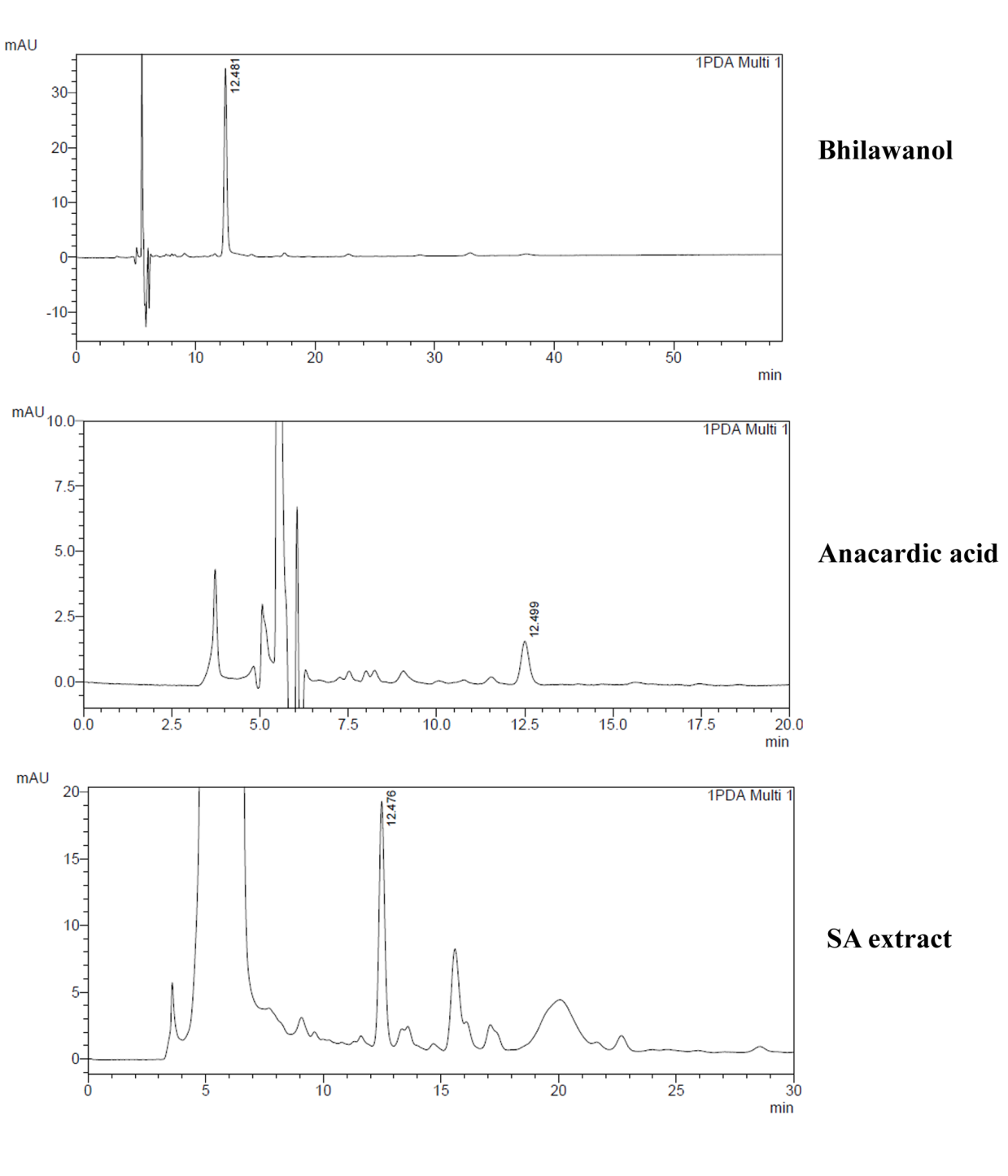


Fig.sup1. Chromatogram of Bhilawanol, anacardic acid and SA extract.
